# Medical management of painful achalasia: a patient-driven systematic review

**DOI:** 10.1093/dote/doae005

**Published:** 2024-01-27

**Authors:** Solange Bramer, Amanda Ladell, Hannah Glatzel, Alan Moss, Majid Hashemi, Giovanni Zaninotto, Stefan Antonowicz

**Affiliations:** General Surgery, Barnet Hospital, London, UK; Achalasia Action, London, UK; Trauma & Orthopaedics, Stoke Mandeville Hospital, Aylesbury, UK; Achalasia Action, London, UK; Upper Gastrointestinal Service, University College London, London, UK; Department of Surgery and Cancer, Imperial College London, London, UK; Department of Surgery and Cancer, Imperial College London, London, UK

**Keywords:** Achalasia, medication, pain, spasm

## Abstract

Achalasia is a rare esophageal disorder characterized by abnormal esophageal motility and swallowing difficulties. Pain and/or spasms often persist or recur despite effective relief of the obstruction. A survey by UK charity ‘Achalasia Action’ highlighted treatments for achalasia pain/spasms as a key research priority. In this patient-requested systematic review, we assessed the existing literature on pharmacological therapies for painful achalasia. A systematic review of the literature using Medline, Embase and Cochrane databases was performed to identify studies evaluating pharmacological therapies for achalasia. Methodological quality of included randomized controlled trials was assessed using the Cochrane Risk of Bias tool. In total, 70% (40/57) of survey respondents reported experiencing pain/spasms. A range of management strategies were reported. Thirteen studies were included in the review. Seven were randomized controlled trials. Most studies were >30 years old, had limited follow-up, and focussed on esophageal manometry as the key endpoint. Generally, studies found improvements in lower esophageal pressures with medications. Only one study evaluated pain/spasm specifically, precluding meta-analysis. Overall risk of bias was high. The achalasia patient survey identified that pain/spasms are common and difficult to treat. This patient-requested review identified a gap in the literature regarding pharmacological treatments for these symptoms. We provide an algorithm for investigating achalasia-related pain/spasms. Calcium channel blockers or nitrates may be helpful when esophageal obstruction and reflux have been excluded. We advocate for registry-based clinical trials to expand the evidence base for these patients.

## INTRODUCTION

Achalasia is a rare esophageal motility disorder characterized by lack of esophageal peristalsis and failure of relaxation of the lower esophageal sphincter (LOS), due to loss of myenteric neurons. The non-peristaltic esophagus may have different manometric patterns (atonic, spastic, pressurized).^1^ Typical symptoms include dysphagia to liquids and solids, regurgitation, weight loss and chest pain. The treatment of achalasia has been focussed on the relief of obstruction, and has classically included laparoscopic cardiomyotomy or endoscopic balloon dilatation(s) as definitive approaches,^2^ or more recently per-oral endoscopic myotomy. Notably, all these treatments are designed to relieve the obstruction only—they do not directly treat the underlying pathophysiology—and these patients will continue to have achalasia regardless of whether the obstruction is relieved. The American College of Gastroenterology recommends that the use of medications (e.g. calcium channel antagonists or nitrates) is limited to achalasia patients who are otherwise not suitable for endoscopic or surgical treatments and have failed to respond to botulinum toxin injections.^3^ The European guidelines on achalasia only support the use of oral pharmacological therapies for recurrent or persistent chest pain after achalasia treatment.^4^

The rarity of achalasia causes a significant challenge in the delivery of clinical trials in this condition. Given this frustration, Achalasia Action,^5^ a registered charity that supports achalasia patients, surveyed its members to generate research priorities. Spasms were reported by the majority of survey respondents, in line with Kalantari *et al.* who showed that patients feel there is a lack of treatments specifically for these symptoms.^6^ There is no recognized definition for spasms related to achalasia, nor how this differs from a spectrum of symptoms including pain, and we have therefore grouped the symptoms using the term ‘pain/spasms’. Achalasia Action asked us to systemically review the literature for the pharmacological management of achalasia, with a specific goal of identifying evidence for the treatment of pain/spasms. Together with a description of the patients’ survey, we provide this review in this paper. There is almost a complete gap in the literature for the evaluation of medical treatment of pain/spasms in patients with achalasia. Further studies are needed to address this unmet need which has been identified as a priority by patients with this condition.

## METHODS

### Patient survey

Achalasia Action, a UK-based registered charity that supports patients with achalasia, distributed a survey to its members to generate research priorities. The survey questions focused on pain/spasms, their frequency, and exacerbating and alleviating factors. The survey author (AL) and the charity (Chair: AM) provided permission to use and publish this data.

### Search strategy

**Fig. 1:**
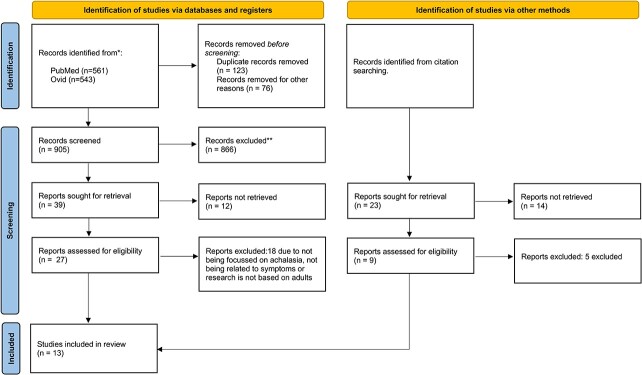
PRISMA flowchart outlining study selection^7^.

PRISMA guidelines^7^ were adhered to for the review strategy, which included independent searches of PubMed, Embase and Cochrane databases by two authors (SB and HG). The following medical subject headings were used: ‘achalasia’ (Mesh), ‘drug therapy’ (Mesh), ‘medical management’ (Mesh), ‘drug treatment’ (Mesh). The terms were combined with AND and OR Boolean operators. The searches were repeated several times, including the final search of 1^st^ June 2023. There was no limit on publication date. Exclusion criteria included no free text available, non-English language, animal subjects, case reports, or non-achalasia human studies. Systematic reviews and meta-analyses were also excluded but their bibliographies were scrutinized for additional references. Titles, abstracts and full-texts were processed using a PRISMA algorithm (Fig. 1). Ultimately, studies which investigated the medical treatment of achalasia were included in the final analysis.

### Data extraction

Two authors (SB and HG) then extracted the following data items; discrepancies were settled with a third author (SA). Data features for extraction included authors, publication date, study type, sample size, assessment of achalasia (manometry, contrast studies, endoscopy), medical treatment (drug, dose, route, duration), non-medical pre-treatment (e.g surgery, endoscopic dilatation), study endpoints (symptomatic, manometric, radiological), side effects and duration of follow-up.

### Quality assessment

The quality of the randomized controlled trials was assessed using the Cochrane risk of bias tool for randomized trials (RoB 2.0). Studies were graded across domains as ‘low risk’, ‘some concerns’ or ‘high risk’, leading to an overall risk of bias.

## RESULTS

### Patient survey

A total of 57 achalasia patients participated in the survey undertaken by Achalasia Action. Not all survey responses were complete. 70% of respondents (*n* = 40/57) reported experiencing pain/spasms. 19% of respondents (*n* = 11/57) did not know whether they were experiencing pain/spasms. The survey did not provide a definition for pain/spasms associated with achalasia and patients described the pain/spasms they experience in different ways, such as ‘pain similar to compression or punch to the chest … particularly during and after meals as food goes down’, ‘permanent feeling of pressure in my upper body’, ‘once I suffer them, it can take quite a few days to get my digestion back again’ and ‘the pain in my chest can be excruciating’. The effect of surgery on symptoms varied between respondents, with 45% (26/57) reporting an improvement, and 31% (18/57) reporting a worsening. Stress was the most frequently reported trigger for spasms (37%, 21/57) while 28% (16/57) of respondents could not identify a cause or trigger for their spasms. Patients reported a variety of techniques to prevent spasms, such as sleeping upright, staying calm, drinking water and dietary changes (e.g. eating little and often or avoiding certain foods). One respondent described taking nifedipine regularly to prevent spasms. Methods to alleviate the pain varied between patients, such as drinking water (most common), deep or slow breathing, massaging/rubbing area, vomiting/retching, and taking medications (paracetamol, codeine, Gaviscon). Full results of the survey can be found in the supplementary materials.

### Systematic review

A total of 13 studies met the selection criteria (Fig. 1)^8–20^. Calcium channel blockers were the most frequently investigated medication, followed by nitrates, and several studies evaluated other drugs. Table 1 outlines the mechanism of action in achalasia and recognized adverse effects of these drugs. Characteristics of the included studies are summarized in Tables 2–4. Seven studies were randomized controlled trials (RCT), and the remainder were uncontrolled clinical trials and case series. Only one study^8^ reported the primary outcome of pain/spasm, and therefore meta-analysis was not performed.

**Table 1:** Medications evaluated for the medical management of painful achalasia

**Drug class**	**Mechanism of action in achalasia**	**Common adverse effects** ^30^
Calcium channel blockers^3^	Reduce intracellular uptake of calcium, hence decreasing muscle contractility, which relaxes the LOS	Headache, dizziness, flushing, peripheral oedema, nausea & vomiting, abdominal pain
Nitrates^3^	Cause the release of nitrous oxide, resulting in relaxation of the LOS	Headache, dizziness, flushing, drowsiness, nausea & vomiting, hypotension
Sildenafil^21^	Inhibits phosphodiesterase type 5, hence increasing cyclic monophosphate levels, which have an inhibitory effect on smooth muscle cells	Headache, dizziness, fluid retention, gastrointestinal symptoms including diarrhea, nausea, night sweats, anxiety, insomnia, cough
Terbutaline^19^	Stimulates beta_2_-receptors in smooth muscle cells which causes relaxation of the smooth muscle	Headache, nausea, arrythmias, palpitations, tremor, hypotension, hypokalaemia, muscle spasms

LOS, lower esophageal sphincter.

**Table 2:** Characteristics of studies evaluating the use of calcium channel blockers in achalasia

**Study**	**Design**	**Sample size (*n*)**	**Previous achalasia treatment (*n*, %)**	**Drug (+/−formulation)**	**Significant reduction in LOS pressure**	**Symptomatic improvement (*n*, %)**	**Effect on pain**	**Side effects** **(*n*, %)**	**Intolerable side effects** **(*n*, %)**	**Follow-up**
Bortolotti 1981^9^	RCT	29	8 (28)	Nifedipine SL	Yes	21 (72)	NR	10	0	6–18 months
Bortolotti 1999^10^	RCS	56	NR	Nifedipine SL	NR	39 (70) at 2 weeks, 13 (22) long-term	NR	unclear	6	Unclear
Coccia 1991^11^	RCT	30	NR	Nifedipine SL	Yes	12 (71)	NR	unclear	1	12–36 months
Gelfond 1982^12^	RCT	15	3 (20)	Nifedipine SL	Yes (46.7%)	8 (53)	NR	2	At least 1	5–14 months
Ghosh 1994^13^	RCS	30^†^	NR	Nifedipine	Yes	Unclear^‡^	NR	NR	NR	N/A
Robertson 1989^14^	Clinical trial	35^§^	10 (29)	Nifedipine SL	No	10 (67%)	NR	3	0	1 month
Traube 1989^15^	RCT	10	3 (30)	Nifedipine SL	Yes (28%)	Significant improvement in number of meals with dysphagia	NR	unclear	1	6 weeks
Triadafilo-poulos 1991^8^	RCT	10	2 (20)	Nifedipine, verapamil	Yes (range 4–43%)	No significant effect	No significant effect	NR	NR	4 weeks
Yasawy 2014^16^	RCS	31^||^	0 (0)^¶^	Nifedipine	NR	0 (0)	NR	NR	NR	4 months

ISDN, isosorbide dinitrate; LOS, lower esophageal sphincter; N/A, not applicable; NR, not reported; RCS, retrospective case series; RCT, randomized controlled trial; SL, sublingual.

^†^Study retrospectively examined 30 patients who had undergone pneumatic balloon dilatation. 12 patients received nifedipine.

^‡^Of patients aged 24–65 years old, five responded to pharmacological treatment, but it is not stated whether this was after nitrates or nifedipine. No patients >65years old responded to pharmacological treatment. All patients required pneumatic dilatation.

^§^Total sample size was 35, of which 15 had achalasia and were the only ones to receive nifedipine, 10 had achalasia and had undergone Heller’s myotomy, 10 were healthy subjects.

^||^Total sample size of the study was 31, of which five received nifedipine.

^¶^No previous endoscopic/surgical treatment.

**Table 3:** Characteristics of studies evaluating the use of nitrates in achalasia

**Study**	**Design**	**Sample size (*n*)**	**Previous achalasia treatment (*n*, %)**	**Drug (+/−formulation)**	**Significant reduction in LOS pressure**	**Symptomatic improvement (*n*, %)**	**Effect on pain**	**Side effects** **(*n*, %)**	**Intolerable side effects** **(*n*, %)**	**Follow-up**
Gelfond 1981^17^	Clinical trial	24	8 (33)	ISDN SL	Yes (65.9%)	19 (79)	NR	8 (33)	0 (0)	2–19 months
Gelfond 1982^12^	RCT	15	3 (20)	ISDN SL	Yes (63.5%)	13 (87)	NR	6 (40)	2 (13)	5–14 months
Ghosh 1994^13^	RCS	30†	NR	ISDN SL	NR	Unclear‡	NR	NR	NR	N/A
Rozen 1982^18^	Clinical trial	15	3 (20)	ISDN SL	Yes (49.6%)	9 (60)	NR	2 (13)	2 (13)	12 months (mean)
Wong 1987^19^	RCT	15	0 (0)	Nitroglycerin SL	Yes	NR	NR	NR	NR	30 minutes

ISDN, isosorbide dinitrate; LOS, lower esophageal sphincter; N/A, not applicable; NR, not reported; RCS, retrospective case series; RCT, randomized controlled trial; SL, sublingual.

^†^Five patients received nitrates. The study retrospectively examined 30 patients who had undergone pneumatic balloon dilatation.

^‡^Of group two patients (24–65 years old), five responded to pharmacological treatment, but it is not stated whether this was after nitrates or nifedipine. No patients >65 years old responded to pharmacological treatment. All patients required pneumatic dilatation.

**Table 4:** Characteristics of studies evaluating drugs other than nitrates or calcium channel blockers

**Study**	**Design**	**Sample size (*n*)**	**Previous achalasia treatment (*n*, %)**	**Drug (+/−formulation)**	**Significant reduction in LOS pressure**	**Symptomatic improvement (*n*, %)**	**Effect on pain**	**Side effects** **(*n*, %)**	**Intolerable side effects** **(*n*, %)**	**Follow-up**
Bortolotti 2000^20^	RCT	14	0 (0)†	Sildenafil	Yes (55.6%)	NR	NR	1	NR	60 minutes
Wong 1987^19^	RCT	15	0 (0)	Terbutaline sulfate, aminophylline	Yes	NR	NR	NR	NR	60 minutes

ISDN, isosorbide dinitrate; LOS, lower esophageal sphincter; N/A, not applicable; NR, not reported; RCS, retrospective case series; RCT, randomized controlled trial; SL, sublingual.

^†^No participants had treatment for their achalasia in the last 6 months.

### Calcium channel blockers

The nine studies evaluating calcium channel blockers (CCB) were published between 1981 and 2014, and included a total of 220 study subjects.^8–16^ Characteristics of these studies are shown in Table 2. Five were randomized controlled trials,^8^^,^^9^^,^^11^^,^^12^^,^^15^ one was a non-controlled clinical trial^14^ and three were retrospective case series.^10^^,^^13^^,^^16^ One study evaluated nifedipine and verapamil,^8^ while all other studies only assessed nifedipine. Follow-up in the studies ranged between 4 weeks and 36 months. In five studies a quarter of participants were pre-treated,^8^^,^^9^^,^^12^^,^^14^^,^^15^ but the authors did not provide subgroup analysis based on prior treatment. Three of the remaining studies did not report whether patients had received prior treatment.^10^^,^^11^^,^^13^

Seven of the nine studies evaluated the effect of CCB on the LOS pressure as their primary endpoint,^8^^,^^9^^,^^11–15^ and all but one found a significant reduction in pressure after CCB.^14^ Five studies identified a symptomatic improvement after CCB use, and this ranged between 53 and 72% of participants.^9–12^^,^^14^ Bortolotti *et al*. reviewed achalasia patients over a 20-year-period.^10^ They stated that 70% of participants continued with nifedipine after two weeks and 22% after follow-up, but did not state the duration of follow-up for individual patients.^10^ Traube *et al.* identified a significant improvement with nifedipine compared to placebo specifically in the frequency of dysphagia during meals^15^ while the remaining two studies found no significant symptomatic improvement after CCB.^8^^,^^16^ Only one study evaluated pain/spasms specifically.^8^ Triadafilopoulos *et al.* identified an at least 50% reduction in chest pain seen in two participants after nifedipine and in three participants after verapamil, but this did not reach statistical significance in this small cohort.^8^

Several CCB studies provide an insight into the duration of improvement seen with CCB and the magnitude of the improvement experienced by patients compared to other treatment modalities. Robertson *et al.* compared symptomatic improvement with nifedipine to pneumatic dilatation and found a greater benefit in patients undergoing dilatation, both in terms of number of participants reporting an improvement and effect size of improvement.^14^ Yasawy *et al*. reviewed the records of 31 patients with new diagnoses of achalasia at their center over 11 years. All patients who received nifedipine (*n* = 5) chose to have pneumatic dilatation.^16^ This is similar to the study performed by Triadafilopoulos *et al.* in which no statistically significant effect was found after CCB, and all participants proceeded to have pneumatic dilatation.^8^ However, half of the participants in this study could not complete the placebo trial due symptoms of achalasia, which did not occur in patients taking nifedipine.^8^ Conversely, Coccia *et al.* stated that pneumatic dilatation and nifedipine were equally effective as 75% of patients after pneumatic dilatation and 71% of patients after nifedipine reported a good to excellent symptomatic improvement.^11^ Ghosh *et al.* undertook a retrospective case series on patients who had previously undergone pneumatic dilatation (*n* = 30), of which twelve patients received pharmacological therapy. Five patients aged 24–64 years old improved symptomatically after pharmacological treatment but it is unclear which drug (nifedipine, nitrates or both) was administered to these patients.^13^ It is therefore not possible to understand which drug this improved clinical response corresponded to. No patients over 65 years old responded to pharmacological treatment. All patients required pneumatic dilatation.^13^ These results suggest calcium channel blockers elicit some improvement over placebo, but not as much as pneumatic dilatation.

Six of the nine studies reported on side effects.^9–12^^,^^14^^,^^15^ Side effects were mild in two studies, with three participants (20%) experiencing mild headache in one study^9^ and 10 participants (34%) experiencing side effects in the other, which included headache, peripheral edema and hypotension (least common).^15^ In four studies, participants withdrew due to side effects.^10–12^^,^^15^ Gelfond *et al.* reported side effects in two (13%) study participants after nifedipine but did not describe the nature of the side effects. One participant did not tolerate nifedipine and therefore underwent pneumatic dilatation and the severity of side effects in the other study participant is unclear.^12^ Three studies only reported the number of participants with intolerable side effects who withdrew from the study, without stating whether other participants also experienced side effects but were able to tolerate the drug and continue with the study.^10^^,^^11^^,^^15^ In these studies, the proportion of participants with intolerable side effects was 3% (*n* = 1/30),^11^ 10% (*n* = 1/10)^15^ and 11% (*n* = 6/56).^10^ Headache was the most common side effect, followed by hypotension.^10^^,^^11^^,^^15^ Triadafilopoulos *et al.* was the only randomized controlled trial on CCB that did not report on the frequency of side effects, or whether the occurrence was significantly different to that in the placebo group.^8^

#### Nitrates

The five studies evaluating nitrates were published between 1977 and 1987 and included 171 participants.^12^^,^^13^^,^^17–19^  Table 3 outlines the characteristics of these studies. Four studies evaluated isosorbide dinitrate, which is a nitrate with a slower onset of action and effects last several hours.^12^^,^^13^^,^^17^^,^^18^ One study investigated nitroglycerin (glycerin nitrate), a nitrate with a rapid onset of action with effects often felt within minutes, but its duration of action is short, ~20–30 minutes.^19^ There were two randomized controlled trials,^12^^,^^19^ two non-randomized clinical trials^17^^,^^18^ and one retrospective case series.^13^ The number of participants included ranged between 15 and 30. One study^8^ did not report whether participants had previously been treated for achalasia and another study^19^ only included treatment-naïve participants. In the remaining three studies, between 20% and 33% of participants had received prior achalasia treatments.^12^^,^^17^^,^^18^ These studies did not compare effects of the drug between pre-treated and treatment-naïve patients.

Four studies investigated the effect of the drug on the LOS pressure as a primary outcome and all found a significant reduction in LOS pressure after nitrate administration.^12^^,^^17–19^ Improvements in esophageal emptying were noted by Gelfond *et al.*  ^17^ and Wong *et al.*^19^ Four studies assessed the effect of nitrates on clinical outcomes, but these were all related to obstruction—such as dysphagia and regurgitation.^12^^,^^13^^,^^17^^,^^18^ The impact of nitrates on pain/spasms was not assessed in any study. Duration of follow-up was very variable in the five prospective studies, with some reporting several months of follow-up (2–19 months), and others just 30 minutes of post-dose observation.^19^

Three studies reported on side effects and all investigated sublingual isosorbide dinitrate (ISDN). In two studies,^12^^,^^18^ a small number of patients stopped taking the drug due to side effects (*n* = 2/15, 13%, in both studies). Overall frequency of side effects was 13% (*n* = 2/15),^18^ 33% (*n* = 8/24)^17^ and 40% (*n* = 6/15),^12^ although Rozen *et al.* did not state whether other participants experienced side effects, in addition to those with intolerable side effects.^18^ Gelfond *et al.* described that seven participants (29%) experienced headaches, of which five had improved clinically after ISDN, and one (4%) had syncope.^17^ Interestingly, two studies noted an improvement in side effects after switching from sublingual to oral tablet formulation of nitrates.^17^^,^^18^ Gelfond *et al.* reported that the participants who improved with ISDN and experienced side effects (*n* = 6), were all able to tolerate the drug after it was switched to oral formulation.^17^ Rozen *et al*. offered oral tablets to every study participant who experienced side effects.^18^

#### Other medications

Two studies were identified on the use of drugs other than nitrates and CCB in achalasia. Both were randomized controlled trials and were published in 1987 and 2000. Study characteristics are shown in Table 4.

Bortolotti *et al.* performed a placebo-controlled trial of sildenafil in 14 achalasia patients.^20^ They found LOS pressure be significantly lower after administration of sildenafil than at baseline or placebo, however they did not evaluate symptom control or the effect of sildenafil on pain/spasms. One study participant developed a headache after sildenafil. There were no other side effects.

Wong *et al.* studied terbutaline sulfate and aminophylline (alongside nitrates, discussed above) and only assessed LOS pressure.^19^ ‘Responders’ were defined as a reduction in LOS pressure $\ge$25% after administration of the drug. There were eight (53%) responders after terbutaline sulfate and four (27%) after aminophylline. Terbutaline sulfate also improved esophageal emptying.^19^ Symptomatic improvement or side effects were not described.^19^

### Assessing the quality of the evidence

The results of the quality assessments of the randomized controlled trials using the Rob 2.0 tool are summarized in Table 5. All studies were at risk of bias. All studies stated that study subjects were randomized but did not explain the method. In two of the included RCTs, this was the only potential source of bias.^15^^,^^20^ Five of the RCTs were randomized controlled crossover trials,^8^^,^^9^^,^^12^^,^^15^^,^^19^ of which four were at high risk of bias. Three of these studies had appropriate wash-out periods. In one study, the different drugs evaluated were administered on consecutive days,^19^ and it is unclear whether this was enough time for the effects of the drugs to wear off. Two studies^11^^,^^19^ did not state whether blinding took place of the patients^19^ or assessors. There was no blinding of study participants in another two studies, of which one study blinded assessors but not patients.^12^ Three studies^8^^,^^9^^,^^11^ were at high risk of bias from missing outcome data by excluding from the analysis patients who did not complete the trial (e.g. patients who withdrew from the trial due to lack of improvement in symptoms or intolerable side effects). Sample sizes in the RCTs were small, ranging between 10 and 30 for CCB,^8^^,^^9^^,^^11^^,^^12^^,^^15^ 15 for both RCTs on nitrates^12^^,^^19^ and the sildenafil RCT had 14 participants.^20^ None of the RCTs reported a sample size calculation, making it difficult for readers to understand whether the sample size was sufficient to identify clinically relevant and significant effects.

**Table 5:** Risk of bias in the randomized controlled trials evaluating drugs in achalasia

	**Drug(s) evaluated**	**Randomization**	**Period and carryover effects**	**Deviations from intended interventions**	**Missing outcome data**	**Measurement of outcome**	**Selection of reported result**	**Overall risk**
Gelfond 1982	Nitrates, CCB	+	-	++	-	-	-	++
Wong 1987	Nitrates, terbutaline sulfate, aminophylline	+	++	++	-	++	+	++
Bortolotti 1981	CCB	+	+	++	++	-	-	++
Coccia 1991	CCB	+	N/A	++	++	++	-	++
Traube 1989	CCB	+	-	-	-	-	-	+
Triadafilopoulos 1991	CCB	+	-	-	++	-	-	++
Bortolotti 2000	Sildenafil	+	N/A	-	-	-	-	+

“-”—low risk, “+”—some concerns, “++”—high risk, N/A—not applicable.

## DISCUSSION

The survey undertaken by ‘Achalasia Action’ highlighted that pain/spasms is a significant issue for achalasia patients. There is no recognized definition for spasms associated with achalasia and survey respondents described the pain associated with spasms in different ways. Numerous techniques for relieving pain/spasms were described, including a minority using medication successfully. Although this survey was likely biased toward those with symptoms, a large number of patients experienced pain/spasms, which supports further research into this issue.

This patient-requested systematic review is the first review to specifically survey the available evidence for the medical management of achalasia and especially achalasia-related pain/spasms, and is the first step in addressing this patient research priority. The majority of studies evaluated were old and of pilot character, especially for nitrates and CCB, with the latest RCTs published in 1987 and 1991, respectively. Only one study assessed the effect of medication on achalasia-related pain/spasms. None have provided focused data on ongoing pain/spasms after the obstruction has been relieved.

Calcium channel blockers were generally effective at reducing LOS pressure at least temporarily, with only one neutral study, suggesting these may be useful in reducing pain/spasms if caused by smooth muscle contraction. Side effects were frequently reported, including therapy-limiting side effects in a minority. Some studies noted an improvement in side effects after switching from sublingual to oral formulation of nifedipine, which allowed patients to continue taking the medication. No studies evaluated different treatment strategies (e.g. continuous vs. intermittent dosing), compared different drug preparations (e.g. sublingual vs. oral), or specifically focused on the pre-treated patient cohort. Moreover, where follow-up was reported, this was generally short, and thus the durability of effects is uncertain. Generally the methodological and reporting quality of the included studies was low, in particular the small sample sizes.

Outside of the achalasia setting, there have been a number of studies investigating the medical management of non-cardiac chest pain, of which a proportion has been ascribed to esophageal pathology. A number of medications have been tested including nitroglycerin,^21^ peppermint oil,^22^^,^^23^ sertraline,^24^ imipramine,^25^ and clonidine,^25^ all with some efficacy and tolerable side effect profile. Other studies have also investigated drugs in other esophageal abnormalities and the drugs showed variable effectiveness, such as peppermint oil in diffuse esophageal spasm,^23^ trazodone for esophageal contractile abnormalities^26^ and sildenafil in hypercontractile esophagus patients.^27^ The diverse nature of the underlying conditions means they were excluded from the present review, however these data provide further opportunities for the medical management of pain/spasm in achalasia.

**Fig. 2:**
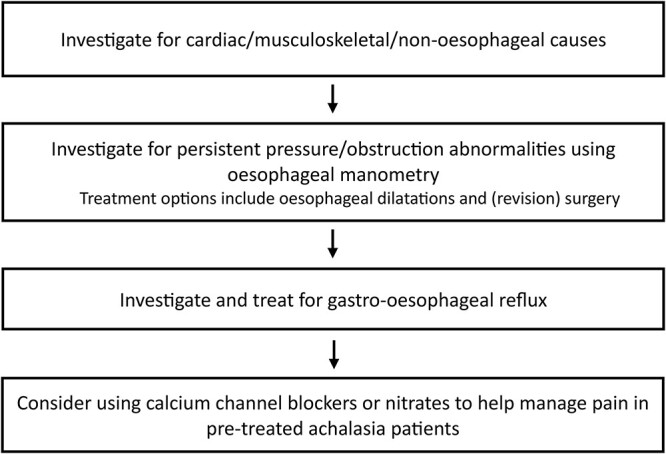
Suggested algorithm for managing recurrent or persistent pain in previously treated achalasia patients.

Achalasia patients presenting with pain, who have previously received achalasia treatment, must undergo a thorough assessment for the cause of their pain. An approach using a step-wise algorithm is suggested, as shown in Figure 2. Cardiac, musculoskeletal and non-esophageal causes of chest pain must initially be excluded and treated accordingly if present. Persistent pressure or obstructive pathology should then be investigated using endoscopy primarily, and then dynamic contrast fluoroscopy and esophageal manometry. An incomplete myotomy can be the cause of persistent symptoms, or esophageal cancer, which has a higher incidence in achalasia.^28^ Persistent achalasia obstruction can be treated with (revision) surgery or esophageal dilatation. In patients with no sign of obstruction, gastro-esophageal reflux should be suspected and investigated, which may require endoscopy or pH impedance monitoring. This can be managed through medications (e.g. proton-pump inhibitors and histamine antagonists) or anti-reflux surgery in refractory cases. When these alternative pathologies have been ruled out, and the pain is felt to be related to achalasia, then medications, such as those described in this review, can be considered as management options, in a step-up approach depending on response and side effects. Further studies focusing on the biomechanics of the esophagus in achalasia are required to understand the pathophysiology of this pain.

The focus of this review was specifically medical management. Endoscopic and surgical management of achalasia has however been extensively studied and reviewed. The use of botulinum toxin has not been included in this review as it is an endoscopic procedure. This procedure is beneficial in elderly patients who cannot undergo more invasive treatments, however this should be used with caution in patients who will subsequently undergo myotomy as it may make the procedure more difficult.

The strengths of this review include the patient-centered approach to the research strategy, which has highlighted a clinical need unmet by the literature. Our review is clearly limited by the design and quality of the current studies, which precluded meta-analysis. Guidelines^3^^,^^4^ and reviews^29^ mention nitrates, calcium channel blockers and other medications as potentially beneficial for the relief of obstruction where other interventions are contra-indicated or unsuccessful. The benefit for achalasia-related pain is essentially not described. We support better adoption of achalasia registries to track patients’ symptoms through their treatment, which would also support the introduction of registry-based clinical trials to expand the drug evidence base in these patients. In the meantime, we suggest an iterative management strategy trialing sublingual preparations of CCBs and nitrates, titrated according to clinical effect and patient side effects, in patients with persistent symptoms in whom further endoscopic or surgical intervention is not indicated.

## CONCLUSION

The patient survey identified that pain/spasms are common and difficult to treat. The findings of this review have shown that the available evidence for the medical management of achalasia is weak and old, with very limited evidence for the management of pain or persistent symptoms after successful relief of obstruction. The best evidence is available for sublingual calcium channel blockers. We advocate for the introduction of an international achalasia registry, from which appropriate clinical trials can be performed to expand the required evidence base.
